# Release mechanisms of major DAMPs

**DOI:** 10.1007/s10495-021-01663-3

**Published:** 2021-03-13

**Authors:** Atsushi Murao, Monowar Aziz, Haichao Wang, Max Brenner, Ping Wang

**Affiliations:** 1 Center for Immunology and Inflammation, The Feinstein Institutes for Medical Research, 350 Community Dr., Manhasset, NY 11030 USA; 2 Center for Biomedical Science, The Feinstein Institutes for Medical Research, Manhasset, NY USA; 3 Department of Molecular Medicine, Zucker School of Medicine at Hofstra/Northwell, Manhasset, NY USA; 4 Department of Surgery, Zucker School of Medicine at Hofstra/Northwell, Manhasset, NY USA

**Keywords:** DAMP, Release, Necrosis, Apoptosis, Exocytosis, Exosome

## Abstract

Damage-associated molecular patterns (DAMPs) are endogenous molecules which foment inflammation and are associated with disorders in sepsis and cancer. Thus, therapeutically targeting DAMPs has potential to provide novel and effective treatments. When establishing anti-DAMP strategies, it is important not only to focus on the DAMPs as inflammatory mediators but also to take into account the underlying mechanisms of their release from cells and tissues. DAMPs can be released passively by membrane rupture due to necrosis/necroptosis, although the mechanisms of release appear to differ between the DAMPs. Other types of cell death, such as apoptosis, pyroptosis, ferroptosis and NETosis, can also contribute to DAMP release. In addition, some DAMPs can be exported actively from live cells by exocytosis of secretory lysosomes or exosomes, ectosomes, and activation of cell membrane channel pores. Here we review the shared and DAMP-specific mechanisms reported in the literature for high mobility group box 1, ATP, extracellular cold-inducible RNA-binding protein, histones, heat shock proteins, extracellular RNAs and cell-free DNA.

## Introduction

Damage-associated molecular patterns (DAMPs) are endogenous molecules which serve as potent activators of the immune system [1]. Examples of DAMPs include nuclear and mitochondrial DNA, RNA, nucleotides and nucleosides, DNA-binding molecules, temperature-shock proteins, and uric acid [1–3]. DAMPs normally reside inside the cell playing diverse roles in homeostasis, but are released to the extracellular space when cells are exposed to stress [1]. Cellular stressors that can lead to DAMP release include a wide array of physical (trauma, radiation), chemical (toxins, osmolarity), metabolic (ischemia/reperfusion), and infectious (viruses, bacteria, protozoa) factors [1, 4, 5]. Once outside the cell, DAMPs are recognized by other cells via their interaction with cellular receptors such as pattern recognition receptors (PRRs), which then upregulate stress response mechanisms that often converge to form a positive feedback loop of tissue injury and inflammation [1]. Indeed, the relevance of DAMPs to various diseases is supported by a number of studies. In sepsis, circulating DAMPs correlate with disease severity and their inhibition has been shown to improve the outcomes in experimental models of sepsis [1]. During cancer different types of DAMPs can promote tumor establishment and progression as well as metastasis [6]. DAMPs are upregulated systemically and locally in patients with autoimmune diseases such as rheumatoid arthritis and their neutralization has shown to prevent the disease progression in animal models [7]. Therefore, DAMP-inhibiting molecules have the potential to significantly attenuate inflammation and in the future may yield a novel class of anti-inflammatory drugs able to finally treat trauma, ischemia/reperfusion injury, sepsis, neuroinflammation, and other pathophysiological conditions irresponsive to existing immune modulatory drugs. Indeed, molecules targeting a number of DAMPs are already in the process of being developed as potential therapeutic agents [8]. At the moment, the predominant strategies to decrease the effects of DAMPs consist of antibody neutralization, competitive antagonism, and enzymatic inactivation [1, 8]. An alternative approach at a more fundamental level is to actively suppress the cellular release of DAMPs. While thorough understanding of the mechanisms of DAMP release may lead to new treatments to attenuate the proinflammatory activity of DAMPs, it might also help to overcome the difficulties we are facing with other forms of immunotherapy such as cytokine removal [9].

**Fig. 1:**
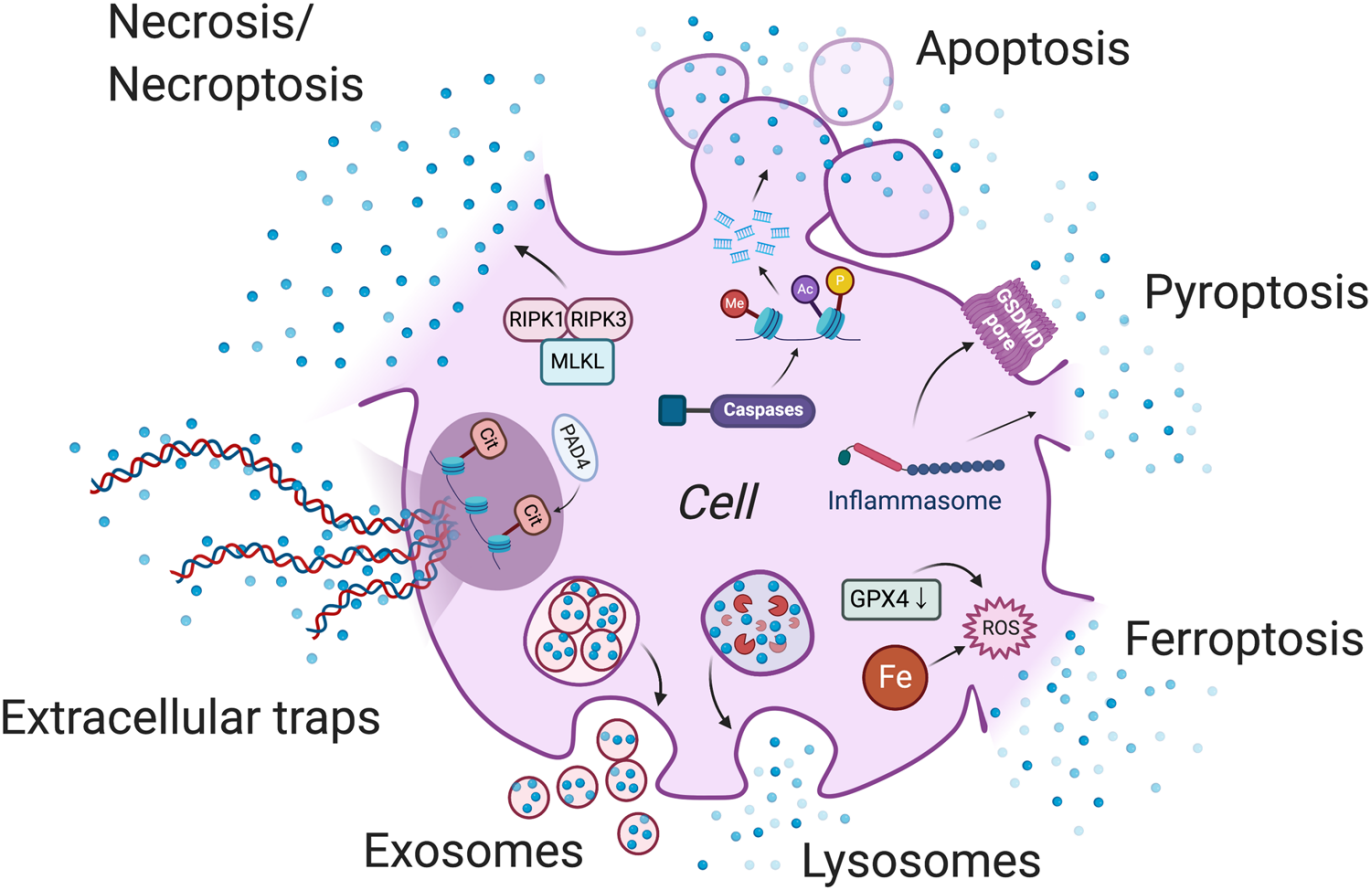
Universal mechanisms of DAMP release. Common mechanisms of DAMP release from the cells are represented by necrosis/necroptosis, apoptosis, pyroptosis, ferroptosis, extracellular traps, secretory lysosomes and exosomes. *RIPK* receptor-interacting serine/threonine-protein kinase, *MLKL* mixed lineage kinase domain like pseudokinase, *GSDMD* gasdermin D, *GPX4* glutathione peroxidase 4, *ROS* reactive oxygen species, *PAD4* peptidylarginine deiminase 4, *Me* methylation, *Ac* acetylation, *P* phosphorylation, *Cit* citrullination

Here we review the mechanisms of DAMP release that have been reported. We first summarize universal mechanisms affecting most if not all DAMPs (i.e., different types of cell death, lysosomal- and exosomal-exocytosis) (Fig. 1). We then outline the mechanisms that have been described for selected DAMPs individually, including high mobility group box 1 (HMGB1), ATP, extracellular cold-inducible RNA-binding protein (eCIRP), histones, heat shock proteins (HSPs), extracellular RNAs (exRNAs) and cell-free DNA (cfDNA), as they have been well-studied among other DAMPs (Table 1).

**Table 1:** Mechanisms and related molecules of DAMP release

DAMPs	Mechanisms of release	Experimental models	Related molecules
HMGB1	Necrosis [16], necroptosis [65, 66], apoptosis [27], pyroptosis [34, 35, 38], ferroptosis [41], lysosomes [47], exosomes [48, 49]	Liver injury [16], infection [66], endotoxemia [38, 48], acute lung injury [34], cancer [107]	JAK/STAT-1 [39], HSP90AA1 [49], XPO1 [49], PKR [67], TLR4/caspase-11/GSDMD [38, 48]
ATP	Necrosis [17], apoptosis [29], pyroptosis [37], lysosomes [50, 69], exosomes [61], channel pores [37, 70, 71]	Sepsis [108], mechanical ventilation [109], hypoxia [110], migraine/stroke [111]	VNUT [69], connexin 43 [70], pannexin 1 [37, 71], TLR/ERK/AP-1 [72]
eCIRP	Necrosis [5], lysosomes [51], extracellular traps [42]	Hemorrhagic shock [51], sepsis [51], acute pancreatitis [42]	GSK3β/CK2 [76]
Histones	Necrosis [18], apoptosis [26], extracellular traps [1], exosomes [52]	Endotoxemia [52], sepsis [112], trauma [113], acute liver failure [114], acute kidney injury [115], acute lung injury [116]	PAD4 [1]
HSPs	Necrosis [19], secretory granules [88], lysosomes [87], exosomes [53], ectosomes [53]	Sepsis [117, 118], acute lung injury [118], cancer [119]	ABC transporter [87]
exRNAs	Necrosis [22], apoptosis [30], exosomes [60], RBPs [101, 102, 120]	Sepsis [121], endotoxemia [122, 123], acute lung injury [122], acute kidney injury [123], liver injury [124]	Ago2/KRAS/MEK/ERK [96]
cfDNA	Necrosis [10, 20], apoptosis [26, 30], extracellular traps [1, 20], pyroptosis [33], ferroptosis [40], exosomes [59], ectosomes [106]	Sepsis [125], acute liver failure [126], acute kidney injury [127], pregnancy [128], cancer [129]	PLA2 [106]

## Universal mechanisms of DAMP release

Different DAMPs share common mechanisms for their release. The release mechanisms can be largely divided into two categories: passive release mainly due to cell death and active release from live cells represented by exocytosis. To be precise, with the exception of necrosis, the other forms of cell death are not entirely passive but rather a regulated process, and some of the mechanisms described under cell death do not always lead to cell death (e.g., NETosis can be suicidal and vital). Thus, it has to be noted that the following categorization is not completely clear-cut and somewhat vague with overlap.

### Passive release (cell death)

DAMPs are well known to be released during different types of cell death. Necrosis is the most common cell death to cause passive release of DAMPs, whereas necroptosis, apoptosis, pyroptosis, ferroptosis and extracellular traps can also contribute to DAMP release. Theoretically, necrosis can cause the release of mixed DAMPs since the cell boundary is lost due to membrane rupture and any cellular components would be released. On the other hand, different forms of cell death can be rather specific to the types of DAMPs as to their release according to the mechanisms. For example, apoptosis leads to the release of nuclear DAMPs following chromatin condensation and DNA fragmentation. Extracellular traps released during NETosis contain DAMPs which are mainly nuclear molecules and antimicrobial enzymes. Conversely, DAMPs can give clues to by which cell death they were released. The fragment length of cfDNA is different according to the type of cell death; cfDNA originating from apoptotic cells is ~ 180 bp due to the fragmentation, while cfDNA released from necrotic cells can be as long as > 10,000 bp [10]. HMGB1 released by pyroptosis via inflammasome pathway is hyperacetylated, which is not seen when it is released from necrotic or apoptotic cells. In addition, the redox state of HMGB1 is in the disulfide form after pyroptosis, in the fully reduced or disulfide form after necrosis, and in the fully oxidized form (sulfonyl HMGB1) after apoptosis [11]. Different DAMPs can be released at the different stages even within the same type of cell death. During apoptosis, ATP is released at the pre-apoptotic stage while HMGB1 is released at the late stage [12, 13]. Besides the preceding DAMPs, it still largely remains elusive and further studies are awaited as to the types and stages of the cell death of their origin.

#### Necrosis and necroptosis

Passive release of DAMPs is typically described to occur as a result of necrosis. Necrosis is commonly caused by extreme chemical or physical insults such as the presence of toxins or trauma, and is characterized by cell swelling and plasma membrane rupture [14]. Tissue ischemia and hypoxia also trigger necrosis by depleting intracellular ATP to unbalance the pump-leak mechanism leading to an influx of Na^+^ and water, which causes cell swelling. Reperfusion can further damage the cells by inducing the generation of multiple oxidants and free radicals [15]. A number of DAMPs have been found to be released by necrosis including, but not limited to, HMGB1, ATP, histones, HSPs, exRNAs, cfDNA, and possibly eCIRP [1, 3, 5, 10, 16–22].

Although cytoplasmic membrane rupture can be uncontrolled accidental (mechanical or chemical) events, it can also be a regulated process governed by specific caspases and kinases. For instance, necroptosis occurs as a result of receptor-interacting serine/threonine-protein kinase 1 (RIPK1) activation followed by RIPK3-dependent phosphorylation of mixed lineage kinase domain like pseudokinase (MLKL) to induce MLKL oligomerization, which results in plasma membrane rupture [14]. DAMP release has been significantly less studied in the context of necroptosis than of necrosis. However, as membrane integrity is lost in necroptosis in a fashion similar to necrosis, theoretically necroptosis also results in the release of DAMPs and other cellular components to the extracellular space [23].

#### Apoptosis

Apoptosis is a regulated cell death without loss of plasma membrane. Morphological features of apoptosis are cytosolic shrinkage, membrane blebbing, chromatin condensation, and DNA fragmentation [14]. The sequential activation of cysteine proteases and endonucleases is the main mechanism of apoptosis, which consists of extrinsic and intrinsic pathway. Extrinsic pathway is induced by caspase-8/caspase-10 or caspase-9 activated by death receptor- or dependence receptor-stimulation, respectively. Intrinsic pathway, on the other hand, is initiated by mitochondrial outer membrane permeabilization followed by the release of mitochondrial proteins, which subsequently causes caspase-9 activation. Both pathways converge on common effector caspases, i.e., caspase-3, caspase-6 and caspase-7, to induce apoptosis [24]. In its early stages, apoptosis is traditionally considered a non-immunogenic form of cell death that prevents the release of intracellular contents because there is no loss of membrane integrity. However, recently it has been revealed that apoptosis can be immunogenic under stress conditions such as chemotherapy or physical modalities. This form of apoptosis is named “immunogenic cell death” (ICD) and is characterized by DAMP release [25]. Indeed, apoptosis has been shown to cause nuclear substances to get exposed at the cell surface and released to the extracellular space and thus act as DAMPs [26]. In the line with its mechanism, nuclear molecules, such as HMGB1, histones, exRNAs and cfDNA as well as ATP have been shown to be released by apoptosis [1, 10, 26–30]. Release mechanisms during apoptosis can be different between DAMPs. Histone release from apoptotic cells is highly associated with DNA fragmentation, which is mediated by caspase-activated DNase/DNA fragmentation factor [28]. ATP has shown to be released from apoptotic cells induced by ER stress in a protein kinase R-like endoplasmic reticulum kinase (PERK) dependent manner [12]. cfDNA and exRNAs have been found in the microparticles released from apoptotic cells [30]. Despite the preceding studies, elucidation of more detailed mechanisms for each DAMP release is awaited in apoptosis.

#### Pyroptosis

Another form of caspase-dependent cell death is pyroptosis, which is induced via the activation of caspase-1 following that of inflammasomes such as NLRP3 or via caspase-4/5/11 activation typically initiated by intracellular LPS [24, 31]. The inflammatory caspase (−1, −4, −5, −11) activation further induces the cleavage of gasdermin D (GSDMD) to promote pore formation in the membrane, allowing the release of intracellular molecules [24, 31]. A recent study has revealed that intracellular protein DDX3X promotes NLRP3 inflammasome activation, which can be inhibited by the induction of stress granules causing the sequestration of DDX3X, thus acts as a live-or-die checkpoint in stressed cells [32]. Pyroptosis is typically known for the release of IL-1ß, though some other DAMPs such as HMGB1, ATP and cfDNA can be released by cells undergoing this type of cell death [1, 33–37]. Even sharing the same signaling pathway, IL-1ß is well known to be released through GSDMD pore whereas a study has shown HMGB1 was released as a result of cell lysis during pyroptosis [38]. In addition, post-translational modifications are required prior to pyroptosis for the release of HMGB1 as described later [39].

#### Ferroptosis

Ferroptosis is a programmed cell death accompanied by iron accumulation and lipid peroxidation. Its morphological features include a loss of membrane integrity, cytoplasmic swelling, swelling of cytoplasmic organelles and moderate chromatin condensation [40]. Intracellular iron accumulation, which can be induced by ferroptosis activators such as erastin and RSL3, causes oxidative stress directly by generating reactive oxygen species (ROS) via the Fenton reaction as well as activating the enzymes responsible for lipid peroxidation and oxygen homeostasis. Glutathione peroxidase 4 (GPX4) is an anti-oxidative enzyme which plays a major role in regulating ferroptosis by preventing lipid peroxidation. Thus, inhibition of GPX4 is a well know mechanism for inducing ferroptosis [40]. Although it is a relatively new concept and less has been elucidated yet, HMGB1 and cfDNA have been regarded to be released by ferroptosis [40, 41]. A study has shown that HMGB1 release during ferroptosis was due to HMGB1 acetylation induced by histone deacetylase (HDAC) inhibition mediated by autophagy [41].

#### Extracellular traps

Neutrophil extracellular traps (NETs) are web-like chromatin-based structures released by neutrophils primarily for pathogen clearance via a regulated process, called NETosis [1]. It is typically described that in the neutrophils undergoing NETosis activated peptidylarginine deiminase 4 (PAD4) citrullinates histones leading to chromatin decondensation accompanied by the dissolution of nuclear and granule membranes. DNA and histones mix with granule-derived antimicrobial peptides in the cytoplasm and are extruded into the extracellular space [1]. Conventionally NETosis was regarded to be a suicidal process leading to cell death (i.e., suicidal NETosis), however later it was found that NETs can also be released from live cells (i.e., vital NETosis) [1]. NETs can be detrimental as they contain not only typical DAMPs, such as histones, cfDNA and eCIRP, but also antimicrobial enzymes, such as neutrophil elastase (NE) and myeloperoxidase (MPO), both of which can directly cause tissue damage [1, 42]. In fact, DNA, histones, NE and MPO are commonly used to identify NETs in experiments or human samples [1, 43]. Though extracellular traps are most clearly observed with neutrophils supported by a numerous amount of studies, similar structures were found to be released from other cell types including macrophages (METs), mast cells (MCETs), eosinophils (EETs) and even B- and T-cells [44–46].

### Active release

Besides the different types of cell death, DAMPs can be released from living or normal cells as well. Many types of DAMPs cannot be released by the typical secretory pathway for proteins consisting of endoplasmic reticulum (ER) and Golgi apparatus as they are nucleotides or proteins without a signal peptide. Well studied carriers of DAMPs during their active release are secretory lysosomes and exosomes, both of which are typically secreted by exocytosis. Even though exocytosis is a regular process even under steady state, increased DAMP release has been observed in this mechanism under stress condition [47–53].

#### Secretory lysosomes

Some DAMPs have been reported to be released via lysosomal exocytosis [36, 47, 50, 51]. The lysosomal import of soluble contents such as hydrolases, which unlike membrane proteins lack sorting signals, is at least in part mediated by their modification with mannose-6-phosphate, which is recognized by mannose-6-phosphate receptor proteins to be loaded into lysosomes [54]. Lysosome secretion can be initiated by the stimulation of cell-surface receptors leading to the increase of intracellular Ca^2+^, which is detected by synaptotagmin (Syt). Syt mobilizes the lysosomes toward microtubule organizing center (MTOC), where the lysosomes associate with a kinesin motor. The motor further transports lysosomes near to the site of secretion, where the lysosomes use actin-based movement to travel to the docking site. Docking and fusion of the lysosomes with the plasma membrane are mediated by RABs and SNARE complexes, respectively [54]. Lysosomal secretion is typical of cells that are stressed and has been identified as one of the release mechanisms of HMGB1, ATP and eCIRP [36, 47, 50, 51].

#### Exosomes

Another mechanism by which DAMPs are released from living cells is via exosomes. Exosomes are extracellular vesicles containing proteins, lipids and nucleic acids to facilitate intercellular communication. Intraluminal vesicles (ILV)—the future exosomes—are formed by inward budding of endosomes to form multivesicular bodies (MVBs) [55]. A well-studied regulator of exosome biogenesis and of the import of its components is the Endosomal Sorting Complex Required for Transport (ESCRT), which works along with its related proteins VPS4, VTA1, ALIX [56, 57]. MVBs are transported to the plasma membrane via interaction with actin, cortactin, microtubule skeleton and RAB proteins [56]. Similar to secretory lysosomes, the MVB docking to the plasma membrane and fusion with it are facilitated by RAB proteins and SNARE complexes, respectively [57]. A large number of DAMPs have been found to be present inside or on the surface of exosomes as we have previously reviewed [58]. Exosomal DAMPs include but are not limited to HMGB1, ATP, histones, HSPs, exRNAs and cfDNA [48, 52, 53, 58–61].

### DAMP receptors

Released DAMPs are recognized by their corresponding receptors including, but not limited to, Toll-like receptors (TLRs), NOD-like receptors (NLRs), receptor for advanced glycation end products (RAGE), triggering receptors expressed on myeloid cells (TREMs) and P2X receptors (P2XRs). Activation of DAMP receptors induces inflammatory response such as cytokine and chemokine production among other effector functions [62]. In addition, the stimulation of the receptors by DAMPs in turn causes DAMP release. For instance, HMGB1 has shown to be released upon NLR and TLR stimulation both passively by cell death and actively by exocytosis [48, 62]. Even though not all the studies about the preceding receptors and DAMP release actually used DAMPs as stimuli of the receptors in the experimental design, theoretically DAMPs may also cause the similar phenomena by acting on the receptors to induce the downstream signaling pathways. Above all, there do exist studies showing that ATP activates P2X7 to induce HMGB1 release via the activation of inflammasome as well as p38MAPK/NF-κB signaling accompanied by ROS accumulation [63, 64].

## Characteristics and release mechanisms for specific DAMPs

### HMGB1

HMGB1 is a nuclear protein capable of binding chromosomal DNA to fulfill its nuclear functions in stabilizing nucleosomal structure and stability and regulating gene expression. As a prototypical DAMP, HMGB1 can be passively released by somatic cells undergoing cytoplasmic membrane destruction due to accidental necrosis or regulated cell death processes such as necroptosis, pyroptosis ferroptosis, or apoptosis [16, 23, 27, 34–36, 41, 65, 66]. In addition, HMGB1 can also be actively secreted by innate immune cells in response to microbial infections. Lacking a leader peptide sequence, HMGB1 cannot be actively secreted through classical ER - Golgi exocytotic pathways. Instead, its active secretion is generally regulated by two signaling steps. The first step, translocation from the nucleus to cytoplasm, is induced by JAK/STAT-1 mediated acetylation of the nuclear localization sequences (NLS) within HMGB1 [39]. The translocation has also shown to be mediated by the interaction with HSP90AA1 (heat shock protein 90 alpha family class A member 1) and XPO1 [49]. The second step, secretion from the cytoplasm to the extracellular space, is dependent on the activation of inflammasomes and caspases as described earlier under pyroptosis [34–36]. The latter step is at least partially controlled by double-stranded RNA-activated protein kinase R (PKR) [67]. A recent study has shown that unlike IL-1ß the release of HMGB1 by inflammasome activation was not through GSDMD pore but due to membrane rupture even though it was indeed GSDMD dependent [38]. In addition to its release by pyroptotic cells, HMGB1 is also released by living cells by exocytosis of lysosomes or exosomes [36, 47, 48]. HMGB1 has been found inside secretory lysosomes and it has also reported to be released via exosomes in a TLR4 and caspase-11/gasdermin D dependent manner [36, 47, 48]. Another study has shown that HMGB1 secretion was induced by autophagy machinery and MVB formation, resulting in its release in the form of exosomes [49].

### ATP

ATP is a nucleotide essential for cellular metabolism, however extracellular ATP works as a DAMP via P2XRs [1]. Dosch et al. recently reviewed the mechanisms of ATP release intensively [68]. Like other DAMPs, ATP can be released passively from damaged tissues and dying cells undergoing necrosis or apoptosis and pyroptosis [17, 29, 37, 68]. Mainly two mechanisms have been reported for active ATP release; exocytosis and channel pores. ATP has been detected inside secretory lysosomes and vesicles and it has been proposed to be released via exocytosis [50, 61, 69]. The vesicular nucleotide transporter (VNUT) protein is responsible for the vesicular storage of ATP [69]. ATP is also exported via the pores of connexin and pannexin hemichannels [37, 68, 70, 71]. Connexins and pannexins are both four-pass transmembrane proteins and are represented by connexin 43 and pannexin 1, respectively. While they are likely to be closed under homeostatic conditions, connexin and pannexin hemichannels open in response to various triggers [68]. The state of connexin 43 is regulated by intracellular calcium concentrations, positive cell membrane voltage changes and phosphorylation of its serine residues. Moreover, the expression of connexin 43 is induced by TLR stimulation followed by ERK/AP-1 signaling [68, 72]. Pannexin 1 channels are opened by mechanical stress, increased intracellular calcium or extracellular potassium, and the cleavage of the C-terminal tail of pannexin-1 proteins induced by activated caspase-3/caspase-7 and caspase-11 during apoptosis and pyroptosis, respectively [37, 68]. To induce a more effective localized release of ATP, pannexin 1 has been reported to translocate to the leading edge of polarized neutrophils or the immune synapse of T-cells [71, 73, 74].

### eCIRP

CIRP is an RNA chaperone protein which plays a role in the regulation of a variety of cellular stress responses. Extracellular CIRP (eCIRP) is a DAMP that perpetuates inflammation and contributes to various diseases [5]. An in vitro study showed that passive release by necrosis might not be a major source of eCIRP, although passive release is a likely important source of CIRP in conditions such as trauma, ischemia-reperfusion injury, and sepsis [5, 51]. Extracellular traps have also shown to be sources of eCIRP [42]. Like HMGB1, CIRP does not contain a signal peptide, thus its release is unlikely to be mediated by ER-Golgi dependent classical pathway. The nuclear to cytoplasm translocation of CIRP requires post-translational modifications such as methylation and phosphorylation [75, 76]. The phosphorylation of CIRP was shown to be mediated by GSK3β and casein kinase II (CK2) [76]. CIRP migrates from the nucleus to cytoplasmic stress granules under certain conditions such as oxidative stress, ER stress, hyperosmotic, and heat shock [75]. Stress granules are RNA-protein complexes that assemble when cells undergo polysome disassembly effectively interrupt protein translation in response to the conditions such as those above. When the offending condition is controlled, stress granules disassemble and translation resumes [77–79]. Stress granules interact with the inflammasome pathway as they inhibit NLRP3 inflammasome assembly by sequestering DDX3X protein [32]. Studies have shown stress granule proteins can be released via extracellular vesicles such as exosomes, though it’s still not clear whether CIRP can be released through the same pathway [80–82]. Exocytosis of secretory lysosomes is also likely to contribute to CIRP release as a study showed CIRP was enriched at the lysosomal compartment of macrophages subjected to hypoxia [51].

### Histones

Histones are components of chromatin in the nucleus together with DNA, but can act as DAMPs by binding to PRRs once they are released to the extracellular space [1]. Histones can be released passively by necrosis like other DAMPs [1, 18]. Upon apoptotic signaling, core histones (H2A, H2B, H3, and H4) and a link histone (H1) undergo post-translational modifications (e.g., H2B phosphorylation at serine 14, H2B acetylation at lysine 15, etc.), which have been reviewed in depth by Füllgrabe et al. [83]. During apoptosis, the modified histones separate from the genomic DNA and translocate to the cytoplasm. The histones protrude from the plasma membrane to be exposed at the cell surface and released to the extracellular space through a mechanism that has not yet been elucidated [26]. One of the main components of NETs, citrullinated histones, mediated by PAD4, are released by the NETosis mechanisms described earlier [1]. Histones can also be secreted actively from living cells via exosomal exocytosis. A study has shown that histones were present on the outer surface of exosomes released from LPS-challenged macrophages and interacted with TLR4 directly [52].

### HSPs

HSPs are a family of molecular chaperones maintaining cellular homeostasis [84]. Extracellular HSPs act as DAMPs and correlate with the severity of several disorders such as sepsis and trauma [85, 86]. HSPs can be released passively by necrosis [19, 53]. HSPs lack a secretory signal, thus are unlikely to be released via ER-Golgi transportation. Proposed mechanisms for active HSP release include secretion via ATP-binding cassette (ABC) transporter in the lysosomal pathway and via secretory granules [53, 87, 88]. Above all, the most accepted mechanism of HSP release is via extracellular vesicles. HSPs are found in two types of extracellular vesicles; exosomes and ectosomes [53]. While exosomes are released by the mechanism described in the earlier section, ectosomes are formed by the outward budding of plasma membrane in response to the increase of cytosolic free Ca^2+^ and are released directly to the extracellular space. Ectosomes are also called microvesicles or microparticles [89]. Different types of HSP family, such as HSP27, HSP60, HSP70 and HSP90, have been found to be released via extracellular vesicles as reviewed by Maio et al. [53]. While HSPs might be located within the vesicle lumen, multiple studies showed HSPs were at least in part present on the surface of the extracellular vesicles, allowing them to interact with surface receptors directly [90–94].

### exRNAs

While some exRNAs are known to have beneficial effect, other act as DAMPs to aggravate inflammation [95]. Though exRNAs can be released by necrosis and apoptosis, they are regarded to be more stable when encapsulated within extracellular vesicles, typically in exosomes, to avoid degradation by RNases in biological fluids such as saliva, breast milk, blood, cerebrospinal fluid, follicular fluid, and urine [22, 30, 60]. The import of exRNAs, specifically miRNAs, to extracellular vesicles is mediated by the association with argonaute 2 (Ago2) regulated by KRAS-MEK-ERK signaling [96]. Other RNA binding proteins (RBPs), such as Y-box protein 1 (YBX1), hnRNPA2B1 and SYNCRIP (hnRNPQ), have also been reported to play a role in the sorting of miRNAs [97–100]. RNAs loaded to the extracellular vesicles are released via exocytosis to become exRNAs. RBPs not only mediate the import but also act as carriers of exRNA in the circulation even without the encapsulation by extracellular vesicles [101, 102]. It is still unknown whether RBP/exRNA complexes in the blood are predominantly released passively by cell death or exported actively via an independent pathway.

### cfDNA

DNA in the extracellular space can serve as a DAMP. Various types of cell death, such as necrosis, apoptosis, pyroptosis ferroptosis, and NETosis are known to release DNA [1, 10, 20, 26, 30, 33, 40]. Its mechanisms of release differ according to the pathogenic condition exemplified by NETosis in sepsis patients and necrotic cells in trauma patients [20]. A recent study showed that cellular senescence was a major determinant of cfDNA kinetics by negatively regulating its release. The elimination of senescent cells through apoptosis recovered cfDNA release [103]. In addition to the passive release by cell death, DNA can be released actively via extracellular vesicles, including exosomes and ectosomes [104]. A study suggests that majority of cfDNA in the blood is present in the form of exosomes, thus avoiding its degradation by nucleases [59]. Besides nuclear DNA, cell-free mitochondrial DNA (cf-mtDNA) also acts as a DAMP [105]. Cf-mtDNA was found in platelet-driven ectosomes along with phospholipase A2 (PLA2). PLA2 is a bactericidal enzyme found to digest the cellular membrane leading to the leakage of mtDNA into the extracellular space [106].

## Conclusions and future prospects

DAMPs represented here by HMGB1, ATP, eCIRP, histones, HSPs, exRNAs and cfDNA can be released by several different active and passive mechanisms such as exocytosis of lysosomes/exosomes, necrosis/necroptosis, apoptosis, pyroptosis, ferroptosis, and extracellular traps. Some mechanisms such as necrosis are shared with more DAMPs, while others are relatively DAMP-specific, e.g., channel pores for ATP.

Despite the detailed mechanisms reviewed here, clearly a lot still remains to be elucidated. A more comprehensive understanding of the mechanisms DAMP release and their regulation will not only enable the design of new investigative tools but may also result in new potential therapeutic approaches to attenuate inflammation and tissue injury and thus to improve the outcomes of pathological conditions associated with excessive DAMP release.

## Data Availability

Not applicable.
